# Conservative Treatment in Stress Urinary Incontinence—Narrative Literature Review

**DOI:** 10.3390/life16010069

**Published:** 2026-01-02

**Authors:** Mircea-Octavian Poenaru, Liana Ples, Cristian-Valentin Toma, Fernanda-Ecaterina Augustin, Romina-Marina Sima, Mihaela Amza, Irina Pacu, Giorgia Zampieri, Andrei Sebastian Diaconescu, Daniela Poenaru

**Affiliations:** 1 Department of Obstetrics and Gynecology, University of Medicine and Pharmacy “Carol Davila”, 020021 Bucharest, Romania; mircea.poenaru@umfcd.ro (M.-O.P.); liana.ples@umfcd.ro (L.P.); romina.sima@umfcd.ro (R.-M.S.); mihaela.amza@umfcd.ro (M.A.); irina.pacu@umfcd.ro (I.P.); giorgia.zampieri@drd.umfcd.ro (G.Z.); 2 Bucur Maternity, St. John Hospital, 040057 Bucharest, Romania; 3 Department of Urology, University of Medicine and Pharmacy “Carol Davila”, 020021 Bucharest, Romania; cristian.toma@umfcd.ro; 4 Department of Urology, Prof. Dr. Theodor Burghele Clinical Hospital, 050013 Bucharest, Romania; 5 “St. Pantelimon” Emergency Clinical Hospital, 021623 Bucharest, Romania; 6 Department of General Surgery, Faculty of Medicine, Carol Davila University of Medicine and Pharmacy, 020021 Bucharest, Romania; 7 General Surgery Department, Fundeni Clinical Institute, 022328 Bucharest, Romania; 8 Department of Rehabilitation, University of Medicine and Pharmacy “Carol Davila”, 020021 Bucharest, Romania; daniela.poenaru@umfcd.ro

**Keywords:** pelvic floor muscle training, non-surgical management, female pelvic floor dysfunction, urinary incontinence rehabilitation, guideline-based therapy, quality of life

## Abstract

Background: Stress urinary incontinence (SUI) is the most common subtype of urinary incontinence in women and significantly their affects quality of life. Aim: The aim of this study was to summarize the current evidence about conservative (non-surgical) treatments for female SUI and outline their clinical applicability. Material and methods: A narrative review was performed using structured research involving medical databases over the last 15 years, including systematic reviews, randomized controlled trials, observational studies and key guidelines regarding pelvic floor muscle training (PFMT), electrical and tibial nerve stimulation, acupuncture, pharmacological therapies, local estrogen, pessaries and bulking agents. Results: PFMT represents the primary first-line therapy, with strongest evidence for reducing leakage episodes and improving quality of life when performed correctly and consistently. Other conservative options (electrical and tibial nerve stimulation, acupuncture, duloxetine, local vaginal estrogen, pessaries and bulking agents) may be efficient for selected patients, but generally they have a weaker or more heterogeneous evidence base. They are considered adjuncts or tailored alternatives when PFMT alone is insufficient, not feasible or not accepted. Conclusions: Conservative management, centered on PFMT, should be offered as initial treatment to most women with mild to moderate SUI, with additional modalities being used selectively according to symptom profile, comorbidities and patient preference. A stepwise, individualized approach can control symptoms in many women and may delay or avoid surgical therapy.

## Simple Summary

Stress urinary incontinence (SUI)—urine leakage during effort, coughing, or sneezing—affects up to one in three adult women and has a major impact on their quality of life, even if many women do not seek help. This narrative review summarizes current evidence on non-surgical (conservative) treatments for SUI, including pelvic floor muscle training, electrical stimulation, tibial nerve stimulation, acupuncture, pharmacological options, vaginal estrogen, pessaries, and urethral bulking agents. Pelvic floor muscle training remains the gold-standard first-line therapy, with strong evidence for reducing leakage and improving quality of life when exercises are correctly taught and maintained over time. Other modalities can be used as adjuncts or alternatives in selected patients, especially when surgery is not desired, or it is contraindicated. Overall, a personalized, stepwise approach starting with conservative measures can control symptoms in many women and may delay or avoid the necessity for surgery, while also improving pelvic health before any eventual surgical intervention.

## 1. Introduction

According to the definition provided by the International Continence Society, urinary incontinence (UI) is described as the “complaint of involuntary loss of urine”. Studies indicate that the prevalence of UI during the postpartum period, ranging from six weeks to one year after childbirth, varies between 10.5% and 63% [1]. The three most common types of UI are stress urinary incontinence (SUI), urgency urinary incontinence (UUI), and mixed urinary incontinence. Stress urinary incontinence is characterized by the involuntary leakage of urine from the urethra during physical exertion, effort, or actions such as coughing or sneezing. This condition is believed to result from increased abdominal pressure. Approximately 20% to 40% of women experience symptoms of SUI; however, prevalence and incidence estimates vary depending on factors such as the specific population studied, particularly in relation to age [2].

The prevalence of UI rises with age, affecting over 40% of women aged 70 and older. In the first year postpartum, approximately one of three women suffers from UI on average (pooled prevalence ~31%, range 10–63%). Since UI is strongly linked to aging, its overall prevalence among women is expected to grow in the future as life expectancy continues to increase [3].

The high prevalence of urinary incontinence observed in the postpartum period should be interpreted considering its considerable impact on physical, psychological, and social functioning, which explains why a relatively large proportion of affected women are bothered enough to seek medical attention. Although, under-recognition and embarrassment still lead many women to self-manage with pads, recent data suggest that awareness of available treatments and the perceived effect on quality of life can drive consultation rates close to 50%, which is higher than the help-seeking figures reported in many general adult populations. From a clinical perspective, this relatively high consultation rate is encouraging, as it creates an opportunity to initiate first-line conservative management—particularly supervised pelvic floor muscle training and lifestyle interventions—at an early stage, potentially reducing long-term burden and the future need for surgery. It is also important to recognize that a subset of women present with dual incontinence, defined as the coexistence of urinary and fecal incontinence, which usually reflects more extensive pelvic floor and sphincter damage. In these cases, focusing solely on stress urinary incontinence is insufficient; instead, a comprehensive, multidisciplinary approach is required, integrating pelvic floor muscle training for both bladder and bowel symptoms, optimization of bowel habits, and, when indicated, specialist assessment for anal sphincter injury in line with international consultation recommendations. Early identification of dual incontinence may therefore guide more appropriate treatment pathways and referral to combined uro-gynecological and colorectal care, with the aim of improving overall continence and quality of life [1,2,3,4,5].

Overactive bladder syndrome is characterized by urinary urgency, typically associated with increased frequency with urinary incontinence, and occurs without the presence of a urinary tract infection [6].

Several factors contribute to the development of urinary incontinence (Table 1). Advancing age is a significant risk factor, as the prevalence of urinary incontinence increases with aging due to weakened pelvic floor muscles and decreased bladder elasticity. Genetic predisposition also plays a role, with studies indicating a hereditary component in some cases. Obstetrical history, including multiple pregnancies, vaginal deliveries, and complicated childbirth, can lead to pelvic floor dysfunction, increasing the likelihood of incontinence. Surgical history, particularly gynecological and pelvic surgeries such as hysterectomy, may also contribute by altering the structural support of the bladder and urethra. Smoking is another contributing factor, as chronic coughing associated with smoking places repeated stress on the pelvic floor muscles. Chronic constipation leads to persistent straining during bowel movements, which can weaken the pelvic support structures over time. Menopause contributes to urinary incontinence due to estrogen deficiency, which results in reduced elasticity and strength of the urogenital tissues. Additionally, participation in high impact sports, such as gymnastics, running, or weightlifting, can increase intra-abdominal pressure, potentially leading to stress urinary incontinence by straining the pelvic floor muscles [7,8].

Urinary incontinence can significantly impact multiple aspects of daily life, affecting physical health, psychological state, social interactions, emotional well-being, and sexual relationships. Physically, it can lead to discomfort, skin irritation, and an increased risk of urinary tract infections. Psychologically, it may cause embarrassment, anxiety, and a loss of self-confidence, leading to social withdrawal. Many individuals with urinary incontinence experience limitations in their daily activities, avoiding travel, social gatherings, or exercise due to fear of leakage [4]. Additionally, the condition can strain intimate relationships, as concerns about odor, hygiene, and unpredictability may lead to decreased sexual confidence and avoidance of intimacy. The cumulative effects of these challenges often result in a significant reduction in overall quality of life, making proper diagnosis and management crucial for affected individuals [9].

The diagnosis of SUI should be comprehensive, involving a detailed medical history, physical examination, and specialized tests. According to the latest protocols in obstetrics and gynecology, as well as urology, a thorough patient evaluation includes a bladder diary, a stress test, and urodynamic studies to assess the severity and underlying causes of incontinence [5].

Because of this burden and the clear role of pelvic floor dysfunction, conservative management strategies—particularly pelvic floor muscle training (PFMT), lifestyle modification and other non-surgical interventions—play a central role in both prevention and treatment of female SUI. The aim of this narrative review is to summarize the current evidence about conservative treatment options for SUI in women, including PFMT, electrical and tibial nerve stimulation, acupuncture, pharmacological therapies, local estrogen, pessaries and bulking agents, and to discuss their clinical applicability within a stepwise, patient-centered management framework.

## 2. Materials and Methods

This narrative literature review included a comprehensive analysis of conservative treatment methods for stress urinary incontinence (SUI). Scientific articles were identified through structured searches of PubMed/MEDLINE, Web of Science, Embase, and the Cochrane Library using combinations of the following descriptors: “stress urinary incontinence,” “female,” “conservative treatment,” “pelvic floor muscle training,” “electrical stimulation,” “tibial nerve stimulation,” “acupuncture,” “duloxetine,” “estrogen therapy,” “pessary,” and “guideline.” The literature search encompassed publications from the last 15 years, and only English-language, peer-reviewed articles were included. Article selection focused on high-quality evidence and authoritative sources specifically addressing non-surgical management of SUI.

The research included systematic reviews and meta-analyses, randomized controlled trials, large observational studies, and current clinical guidelines pertaining to SUI. To meet the eligibility criteria, sources needed to be published in peer-reviewed journals indexed by ISI (Impact Factor > 2) or be high-impact guideline publications in the 2010–2025 timeframe. We considered level 1 evidence (e.g., Cochrane reviews, RCTs) and key narrative reviews or guideline documents that synthesize evidence. Studies were excluded at title/abstract or full-text level if they (i) did not focus on female SUI or conservative management, (ii) were case reports, small uncontrolled series or non-peer-reviewed material, or (iii) were superseded by more recent systematic reviews or guidelines addressing the same question.

**Figure 1:**
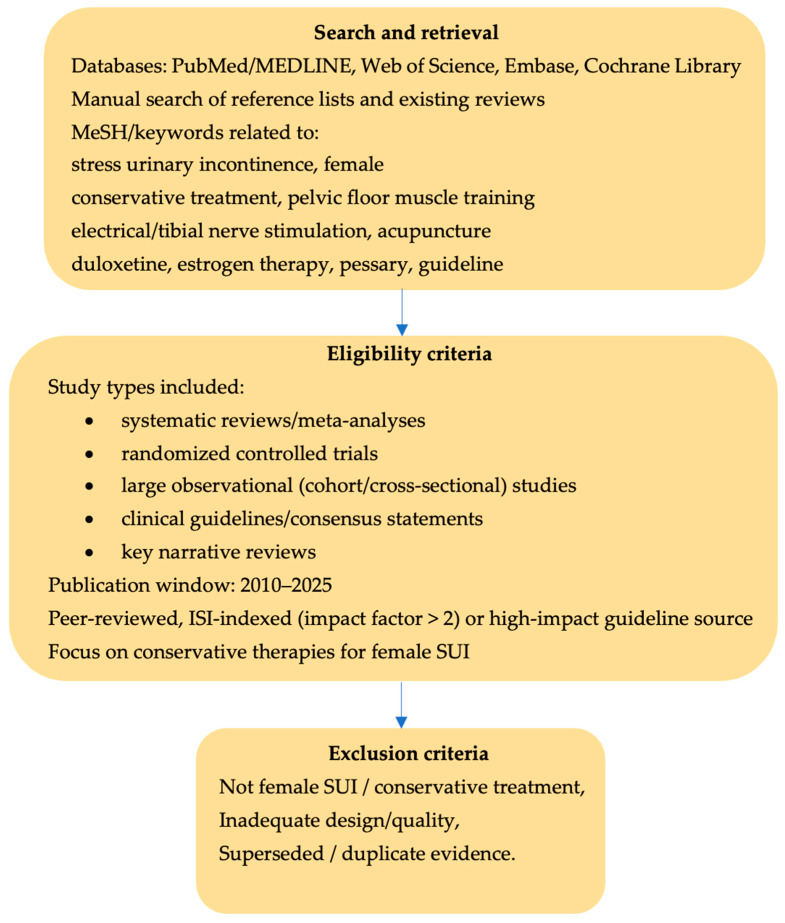
Literature search and study selection for this narrative review. The flow diagram summarizes database searches, screening and full-text assessment, with exclusion of studies that did not address female SUI or conservative treatment, did not meet design/quality criteria (e.g., small uncontrolled series, case reports) or were superseded by more recent high-level evidence.

A number of 180 publications were initially retrieved. After screening titles/abstracts and removing duplicates, 90 full-text articles were assessed for eligibility. Ultimately, 62 sources met inclusion criteria and were incorporated into this review, comprising 3 systematic reviews/meta-analyses, 14 randomized controlled trials, 9 cohort or cross-sectional studies, 4 clinical practice guidelines or consensus statements, and 14 narrative or review articles that provided additional context (e.g., prevalence, quality of life impact, or mechanistic insights). The search and selection process, summarized in Figure 1, was designed to be transparent and structured, but the review was not conducted as a full systematic review. Data from these sources were extracted and organized by intervention category. Given the narrative (non-meta-analytic) nature of this review, we synthesized findings qualitatively. Where applicable, we report representative outcomes (e.g., risk ratios, cure rates) from high-level evidence to illustrate the efficacy of treatments. The strength of evidence is described using guideline-like terminology (e.g., Level 1 evidence for RCTs/meta-analyses) to emphasize quality. Any conflicting evidence or areas with sparse data are explicitly noted. Given that this is a narrative review rather than a formal systematic review or meta-analysis, no structured, study-by-study risk-of-bias assessment tool was applied. Instead, priority was given to high-level evidence such as systematic reviews, randomized controlled trials and major clinical guidelines, while data from smaller or lower-quality studies were included primarily for contextual purposes and are interpreted with appropriate caution.

This integrative methodology enabled the narrative review to capture both the breadth of conservative SUI therapies and the depth of evidence supporting each. By combining rigorous data from recent studies with expert guideline perspectives, we aim to present a balanced and clinically relevant review. The subsequent sections detail the findings for each management approach, followed by an overview of surgical options and a discussion putting the evidence into practice.

## 3. Results and Discussion

The current narrative review synthesized evidence regarding conservative therapeutic approaches for stress urinary incontinence, addressing their mechanistic foundations and clinical efficacy. Pelvic floor muscle training emerged as the gold standard, demonstrating superior long-term outcomes with sustained patient compliance. Electrical stimulation and posterior tibial nerve stimulation offered viable alternatives for patients with inadequate response to standard rehabilitation protocols. Acupuncture and pharmacological interventions presented adjunctive benefits, though evidence remains heterogeneous. Integration of multimodal conservative strategies may optimize treatment outcomes while minimizing invasive surgical procedures.

### 3.1. Pelvic Floor Muscle Training

Pelvic floor muscle training (PFMT), also known as Kegel exercises, is the first-line therapeutic approach to SUI. It prevents urine leakage in two ways: applying pressure on the urethra and providing support for the bladder neck. An intentional and effective pelvic floor muscle contraction (in a cranial and forward direction) prior to and during an effort clamps the urethra, increases urethral pressure and prevents urine leakage. There is some evidence that in healthy continent women, the activation of the pelvic floor muscles before or during physical exertion is an automatic response that does not require conscious effort. In the case of SUI, performing a strong, well-timed pelvic floor muscle contraction may actively prevent urethral descent during an intra-abdominal rise in pressure. Pelvic floor muscles support the bladder neck and limit its downward movement during effort. A toned pelvic floor muscle permanently elevates the levator plate to a higher position inside the pelvis and contributes to leakage prevention [10].

Randomized trials have shown that teaching pelvic floor exercises during pregnancy reduces the risk and severity of SUI in late pregnancy and the early postpartum period, particularly in primiparous women [11,12,13].

Training programs usually involve instruction by a clinician (e.g., physiotherapist or nurse) to ensure correct pelvic floor muscle isolation and technique. Women are typically advised to perform daily exercise sessions over at least a 3–6-month period. A common regimen is 8–12 maximal contractions held for 6–8 s each, repeated 3 times per day. In practice, protocols vary; some incorporate rapid (“flick”) contractions in addition to sustained holds, and positions can range from supine to upright as training progresses. Supervision level and adjuncts differ as well. Supervised, clinic-based PFMT (with periodic follow-up) is generally more effective than unsupervised home exercise, presumably due to better technique and motivation. Group-based training classes can be as effective as individual sessions.

A meta-analysis, originally published in 2001 and updated in 2018 [14], evaluated 31 trials involving 1817 women to assess the effectiveness of pelvic floor muscle training (PFMT) for urinary incontinence (UI). The findings showed the following:

- Symptom resolution: women with stress urinary incontinence (SUI) undergoing PFMT were eight times more likely to achieve symptom relief compared to those receiving no treatment or inactive interventions (56% vs. 6%; RR 8.38, 95% CI 3.68 −19.07; 4 trials, 165 women; high-quality evidence).
- Improvement in symptoms and quality of life (QoL): PFMT significantly improved UI symptoms (7 trials, 376 women; moderate-quality evidence) and QoL (6 trials, 348 women; low-quality evidence) compared to controls.
- Reduction in leakage episodes: PFMT reduced daily urine leakage by an average of one episode (MD −1.23; 95% CI −1.78 to −0.68; 7 trials, 432 women; moderate-quality evidence).
- Less urine loss on pad tests: Women in PFMT groups exhibited significantly reduced urine loss in short-duration (up to one hour) pad tests.
- These results highlight PFMT as a cost-effective conservative treatment for SUI [14].

A randomized controlled study on 44 patients divided into 2 groups included in 12 week-training program in supine versus supine plus upright position found similar results. Overall, the trial reported that 41% of the women experienced a 100% reduction and 20.5% experienced at least a 75% reduction in the number of SUI episodes per week, irrespective of the position of training [15]. The conclusion is that various positions of training (supine, supine and standing) offered no difference for outcomes [16].

The number of vaginal deliveries was documented to play a role in the apparition of SUI, especially in menopause, through various mechanisms (stretching of the birth canal tissues, damage to the levator ani muscle and the visceral pelvic fascia [17], damage to the pudendal nerve in the second phase [18]. A randomized controlled trial on 150 patients compared to a group that received PFMT and transversus abdominis (TrA) training with a group with PFMT alone, with a stratification of patients according to the number of deliveries (fewer than 3 and 3 or more). After 12 weeks of 4 weekly sessions, the improvements were significant only in the subgroup with fewer than 3 deliveries and for the combined training (PFMT + TrA) [19].

Weighted vaginal cones represent a conservative method to train the pelvic floor muscle. The devices are inserted into the vagina and pelvic floor is contracted to prevent them for falling out. Available data suggest that weighted vaginal cones offer superior benefit compared with no active intervention in women with SUI, and their efficacy appears broadly comparable to pelvic floor muscle training and electrical stimulation. However, this interpretation should be regarded as tentative until it is confirmed by larger, high-quality clinical trials [20].

A randomized controlled, cross-over trial on 70 patients compared to an intervention group that received fitness training and PFMT for 12 weeks, twice per week followed by a daily home-based program with a control group with no intervention. After three months, the intervention group improved significantly BMI, walking speed, adductor muscle strength, urine leaking parameters and psychosocial status. There was a positive correlation between urine leaking improvement and walking speed, with a possibility that an increase of 10% or more in walking speed may lead to improvement in SUI, although the mechanism is not clear. Furthermore, there was a positive correlation between BMI and symptoms of SUI, with a cure rate for urine leakage episodes significantly higher in patients whose BMI decreased. The data suggests that a decrease in BMI of 5% or more may lead to improvement of SUI. No correlation was found for adductor muscle strength and urine leaking evolution [21].

Adherence remains a major challenge in PFMT. Symptom improvement correlates strongly with exercise consistency and long-term practice of the exercises. Unfortunately, many women cease to practice them over time. Dropout rates of 15–45% are seen in PFMT programs, often within the first few months. Common barriers include busy schedules, forgetting, uncertainty about technique, or lack of perceived early benefit. Strategies to improve adherence include regular follow-ups (in person or via phone/apps), written exercise schedules or reminders, and emphasizing that benefits accumulate gradually (often 4–6 weeks before noticeable improvement, with maximal gains by 3–6 months) [22].

A large multicentric randomized controlled trial (600 women) further reinforces the role of supervised, protocolized PFMT as the cornerstone first-line therapy for SUI: both groups, with and without EMG biofeedback, achieved similar improvements in urinary symptoms, quality of life and pelvic muscle strength at 6, 12 and 24 months, with cure in about 8% and clinically relevant improvement in approximately 60–63% of patients in each arm. These results support the current grade A recommendation for PFMT in international guidelines [17] and indicate that EMG biofeedback should be viewed as an optional adjunct rather than a mandatory component of treatment, since it does not systematically improve outcomes but entails additional costs; PFMT can therefore be effectively delivered in supervised individual or group settings, as well as through structured home-based programs [23,24].

**Figure 2:**
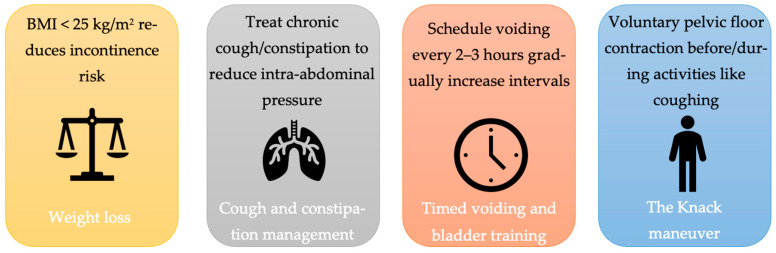
Key lifestyle and behavioral interventions that can be combined with PFMT to reduce stress urinary incontinence episodes.

PFMT is often combined with other conservative measures (Figure 2). Weight loss in overweight patients can significantly reduce SUI episodes—even a 5–10% reduction in body weight is associated with measurable improvement in leakage. A notable trial found that a structured weight loss program (diet plus exercise) led to a 70% reduction in weekly incontinence frequency compared to controls [25]. Therefore, clinicians should counsel SUI patients on weight management (Level 1 evidence supports this, particularly if BMI is more than 30). Management of chronic cough (e.g., smoking cessation to eliminate smoker’s cough) and treatment of constipation can also mitigate SUI trigger [25]. Timed voiding and bladder training techniques, while mainly used for urgency incontinence, may help some SUI patients by preemptively emptying the bladder before physical activities. Additionally, teaching the “Knack” maneuver—a well-timed pelvic floor contraction just before and during moments of increased abdominal pressure (like a cough)—can suddenly reduce stress leakage and is a useful skill for patients to employ in daily life [25]. This maneuver essentially applies PFMT principles in real-time during stressful events.

In summary, PFMT is a highly effective, low-risk therapy for SUI and is widely endorsed as the front-line conservative treatment in major international guidelines and expert consensus documents [14], such as the National Institute for Health and Care Excellence (NICE) and the American Urological Association (AUA) [26,27]. Success requires proper instruction and sufficient duration/intensity of training, as well as strategies to maintain long-term compliance; when implemented appropriately, up to 50–70% of women experience appreciable symptom reduction or cure [28], and preoperative PFMT may also enhance postoperative continence outcomes.

### 3.2. Surface and Intravaginal Electrical Stimulation

Electrical stimulation (ES) of the pelvic floor is a conservative therapy that has been used alone or as an adjunct to PFMT for SUI. The rationale is that low-voltage electrical current, delivered via vaginal or surface electrodes, can induce reflex contractions of the urethral and pelvic floor muscles, strengthening them over time and improving urethral support and closure pressure. Electrical stimulation (ES) can also enhance patients’ awareness of the correct muscles to contract, potentially aiding PFMT learning. Several types of electrotherapies have been studied:

1. Intravaginal electrical stimulation: A probe electrode is inserted into the vagina to directly stimulate the pelvic floor musculature and periurethral tissue. Typically, intermittent, low-frequency (~20–50 Hz) pulses cause muscle contractions. Sessions last ~15–30 min and are carried out a few times per week for 6–12 weeks.
2. Transcutaneous electrical nerve stimulation (TENS): Surface pad electrodes on the skin (e.g., over sacral nerves or pudendal nerve regions) deliver current through the skin to target pelvic floor nerves. This is less focal than vaginal ES but noninvasive.
3. Extracorporeal magnetic innervation (ExMI): Although not electrical current per se, ExMI uses a magnetic field (usually via a chair device) to induce pelvic floor muscle contractions. It is often classed alongside electrical therapies and will be discussed here for convenience.

Intravaginal ES is usually administered with the patient at rest. Typical stimulation parameters for SUI are a 10–50 Hz frequency, pulse width ~0.5 milliseconds, ramping up to a tolerable intensity that induces a contraction, with on/off cycling (e.g., 5 s on, 10–15 s off). Sessions last about 20 min. Most devices are designed for home use after initial instruction. Surface (TENS) treatments target nerves like S2–S4 (sacral nerve roots) or the pudendal nerve; for example, electrode placements on the lower abdomen and buttocks can stimulate the pelvic plexus. Treatments are typically carried out 2–3 times weekly for 8–12 weeks. ExMI devices (e.g., the “magnetic chair”) allow the patient to sit fully clothed while a focused pulsing magnetic field induces pelvic floor contractions; a standard protocol is twice weekly sessions for 6–8 weeks [29].

Electrical stimulation appears to have some benefit over no treatment, but its added value compared to PFMT is debatable. A 2017 Cochrane review analyzed non-implanted electrical stimulation for SUI across 56 trials. It concluded that ES was probably better than no treatment or sham in achieving cure or improvement of SUI (pooled cure/improvement rates ~60% with ES vs. ~34% with sham). However, evidence was insufficient to determine if ES was any better or worse than PFMT alone. In fact, multiple studies suggest that PFMT yields similar or superior outcomes to ES [30]. A new 2025 systematic review by Lunardi et al. compared ES versus PFMT in women with SUI (7 trials, 411 women) and found no significant advantage of ES over PFMT in reducing leakage episodes or improving QoL. Both approaches led to symptom improvement, but ES did not outperform pelvic exercises—implying that the active patient-driven training is the critical element for success. Consequently, the review authors concluded that ES should not be considered superior to PFMT, and routine use of ES as first-line therapy is not justified [22]. Similarly, an earlier RCT found no difference in SUI outcomes between a group receiving ES plus PFMT versus a PFMT-alone group [31].

In terms of tolerability, ES is generally safe. Some women report discomfort with the vaginal probe or muscle fatigue during stimulation. Skin irritation can occur with surface electrodes. Care is needed in patients with pacemakers or arrhythmia—most guidelines contraindicate intravaginal ES in women with pacemakers due to theoretical risk of interference. ES is also contraindicated in pregnancy. Otherwise, adverse events are few [31].

Neuromodulation, specifically electrical stimulation techniques such as sacral neuromodulation (SNM) and peripheral tibial nerve stimulation (PTNS), is an established treatment for overactive bladder (OAB) and for the urgency component of mixed urinary incontinence, rather than for pure stress urinary incontinence (SUI). SNM involves minimally invasive electrode placement at the third sacral root (S3), connected to a pulse generator that modulates bladder activity by suppressing afferent signaling and enhancing spinal reflexes, whereas PTNS and its non-invasive counterpart, transcutaneous tibial nerve stimulation (TTNS), stimulate the tibial nerve near the ankle to reduce bladder overactivity symptoms. Clinical evidence shows that, in patients with refractory OAB or mixed UI, these neuromodulation methods effectively decrease urinary urgency, frequency and urge incontinence episodes [32,33]; TTNS may offer greater comfort and ease of application compared to PTNS. Overall, neuromodulation should be considered a reversible, safe option for the OAB/urgency component when conservative measures and pharmacotherapy have failed, while SUI itself continues to be managed primarily with PFMT and other stress-focused conservative or surgical treatments [34].

### 3.3. Acupuncture

Stimulating the pudendal acupoint nerves through acupuncture can enhance pelvic floor muscle contractions, leading to muscle strengthening and better urinary control. A 2021 meta-analysis from China, which included eight studies (607 patients), found that therapies such as pelvic floor muscle training, electrical stimulation, and pharmacotherapy effectively reduced urine leakage (as measured by the urine pad test) and improved ICIQ-SF scores in the treatment of urinary incontinence in middle-aged and elderly women. However, the effect of acupuncture was found to be more significant. The main weakness of this meta-analysis is the lack of studies on larger population cohorts, limiting its statistical significance [35].

A 2023 meta-analysis of 10 studies found that electroacupuncture may improve stress urinary incontinence based on clinical outcomes. Acupuncture techniques analyzed included body acupuncture, hand acupuncture, Japanese meridian acupuncture, scalp acupuncture, fire needle acupuncture, auricular acupuncture, Sham acupuncture, laser acupuncture, elongated needle acupuncture, and electroacupuncture. Electroacupuncture is a treatment that combines traditional acupuncture with electrical stimulation, where electrodes from an electrical stimulator are attached to the acupuncture needles. Sham acupuncture is an inactive procedure designed to mimic real acupuncture in a clinical trial to serve as a control group. It involves techniques like using blunted needles that do not penetrate the skin, or penetrating the skin at non-acupoint locations, to create a similar looking but physiologically inert treatment. This helps researchers isolate the specific effects of true acupuncture from placebo or other non-specific effects. The study included women with SUI, UUI or mixed UI treated with acupuncture. Control groups received sham/placebo treatments, pharmacotherapy, no treatment, pelvic floor muscle training, or unrelated exercises. Outcomes were assessed through bladder diaries, pad tests, OAB symptom scores, continence recovery, urge accidents, UI episodes, and urine pad usage. Electroacupuncture led to a notable reduction in urinary incontinence episodes and pad usage compared to sham treatment. Similarly, body acupuncture decreased urge incontinence incidents, while laser acupuncture reduced urgency symptoms. The de qi sensation—characterized by warmth and tightening—is thought to enhance acupuncture’s therapeutic effects [36]. A meta-analysis of two moderate-quality studies found that electroacupuncture significantly improved stress urinary incontinence (SUI) compared to sham treatment. Women in the electroacupuncture group reported fewer UI episodes and a reduced need for urine pads [37,38].

Acupuncture (including electroacupuncture) is postulated to improve SUI by enhancing the contraction strength of pelvic floor muscles and modulating neurological reflexes that affect the bladder and urethra. A recent meta-analysis in 2023 by Jiang et al. pooled 10 studies (largely Chinese trials) and reported that electroacupuncture was associated with fewer daily incontinence episodes and pad usage compared to sham treatments. It also suggested that combining acupuncture with PFMT yielded better outcomes than PFMT alone [39].

However, it is important to note that many included trials were small and of moderate quality; the overall evidence grade was low. Cochrane reviews (2013, 2022) have previously remarked that due to methodological limitations of acupuncture studies, the true benefit remains uncertain [39]. Thus, while acupuncture may provide some improvement (and is relatively low risk), it cannot be considered a first-line or standalone therapy for SUI at this time. Clinicians should ensure that patients pursuing acupuncture also engage in proven therapies like PFMT. If used, acupuncture should be performed by qualified practitioners, and electroacupuncture (where mild electrical current is applied through needles) appears more effective than manual acupuncture in the limited data available. Further high-quality RCTs are needed in this area—especially outside of East Asian populations—to validate efficacy and standardize treatment protocols. Until then, acupuncture might be offered as an adjunct for interested patients, with the caveat that evidence is not as strong as for conventional treatments (Level of evidence: low) [39].

### 3.4. Pharmacological Treatment of Stress Urinary Incontinence

Pharmacological treatment of urinary incontinence in women has advanced significantly over the past decade, offering multiple therapeutic options tailored to the type of incontinence and patient profile.

Duloxetine, belonging to the class of serotonin and norepinephrine reuptake inhibitors (SNRIs), is currently the only drug approved in the European Union for the pharmacological treatment of SUI. It acts by enhancing the descending sympathetic pathway that controls the urethral sphincter, thereby increasing its somatic motor activity during the bladder filling phase [40,41]. Multicenter randomized trials have shown a significant reduction in incontinent episodes and improvement in quality of life compared to placebo [42]. A study published in BJU International reported a positive response rate exceeding 60% in women treated with duloxetine [43]. However, the use of duloxetine is limited by frequent adverse effects, including nausea, insomnia, and fatigue, leading to a high treatment discontinuation rate [44]. Up to 20–30% of women discontinue the drug due to side effects such as nausea, dry mouth, insomnia, fatigue, and constipation [45]. Post-marketing surveillance also raised concern about rare serious adverse effects (e.g., suicidal ideation), leading the FDA not to approve duloxetine for SUI in the US. A 2016 meta-analysis weighed the modest benefits against the relatively high dropout and concluded that for most women, “the harms of duloxetine outweigh the benefits” [46]. Indeed, some authors recommend avoiding duloxetine for SUI except perhaps in patients who also suffer from depression or fibromyalgia (where duloxetine could address both) [46]. Current practice patterns reflect this caution—duloxetine is underutilized and often reserved for those who either cannot undergo surgery or want to delay surgery with a medication trial. When used, it is critical to counsel patients on side effects and start with a low dose titration (to improve tolerability). In summary, duloxetine can be efficacious for SUI in some women, but because of side effects, it should be considered an optional adjunct rather than a mainstay (Evidence Level 1 for efficacy, but strong recommendation against routine use in many guidelines due to safety/tolerability [46].

An emerging pharmacologic agent is TAS-303, also selective norepinephrine reuptake inhibitor, acting on urethral sphincter muscle tone. Preclinical and phase II clinical studies have shown a promising efficacy and safety profile, with fewer adverse effects compared to duloxetine [47]. Although not yet widely approved, TAS-303 may represent a viable alternative for patients who do not tolerate existing treatments.

No other systemic drugs have robust evidence in SUI. Agents like alpha-agonists (pseudoephedrine, midodrine) can increase urethral tone but have even weaker supporting data and more cardiovascular side effects, so they are not commonly recommended.

### 3.5. Local Estrogen Therapy

For postmenopausal women with SUI and signs of genitourinary syndrome of menopause (GSM, formerly atrophic vaginitis), topical estrogen can be a useful adjunct. Topical (vaginal) estrogen (creams, pessaries, or rings) improves urethral epithelium vascularity and thickness and may enhance periurethral collagen support. While estrogen alone is not a potent treatment for SUI, it can alleviate concomitant urgency and urinary frequency symptoms and may modestly improve continence in hypoestrogenic women [48].

A 2025 meta-analysis of 17 studies (2111 patients) found that vaginal estrogen therapy significantly improved all evaluated lower urinary tract symptoms, including a reduction in stress incontinence prevalence (pooled OR ~0.12 vs. baseline). Importantly, this analysis supports current guidelines that vaginal estrogen should be offered to menopausal women with UI symptoms and vaginal atrophy (Recommendation Grade B) [49]. In practice, adding a nightly or twice-weekly estradiol or conjugated estrogen vaginal dose to a regimen of PFMT might improve tissue responsiveness and continence outcomes.

The Women’s Health Initiative trials over a decade ago unexpectedly noted higher rates of incontinence in women on systemic estrogen vs. placebo, suggesting that systemic hormone replacement may adversely affect continence mechanisms. By contrast, low-dose local (vaginal) estrogen produces mainly local effects on the urethral and vaginal epithelium with minimal systemic absorption, which likely explains why it can modestly improve stress and urgency symptoms in hypoestrogenic women while systemic estrogen may worsen or fail to improve SUI [50].

### 3.6. Conservative Aids and Devices

A variety of mechanical devices can help manage SUI. The most established are vaginal pessaries designed for incontinence. Continence pessaries (e.g., ring with support, incontinence dish) position the urethra and bladder neck in a more retropubic, kinked orientation and/or apply compression to the urethra to prevent leakage during stress events. Many women with SUI and maybe coexisting mild prolapse can benefit from a well-fitted pessary. Case series report that around 50–70% of women can achieve satisfactory continence improvement with an incontinence pessary, at least in the short term [25]. A notable RCT (the ATLAS trial) found that a pessary was about as effective as a structured behavioral therapy program after 3 months (improvement in ~40% vs. 49%, respectively; not statistically different). Patient satisfaction in one year was also similar between pessary and PFMT groups [51].

These findings support offering a pessary as an initial treatment choice, especially for women who either cannot perform PFMT (or do not improve with it) or prefer a device. Pessaries have minimal risks—some vaginal discharge or ulceration in a minority that usually resolves with cleaning and estrogen cream. The Dutch guidelines actually suggest giving women with SUI the choice between PFMT and pessary as first-line management. Clinical experience indicates pessaries work best in SUI patients with some degree of anterior vaginal wall support defect (where the pessary can coapt the bladder neck). It is important to have the pessary fitted by an experienced provider; sometimes multiple sizes/types are tried to find the best result. Once fitted, a patient can be taught to manage it (remove, clean, replace) if able, or she can have periodic clinical follow-ups for maintenance [25].

Newer disposable intravaginal devices are also on the market—for example, a tampon-like single-use device (Impressa^®^) that women can insert during activities. A 2022 RCT comparing a traditional pessary vs. the Impressa device found both significantly reduced leakage and had comparable efficacy and satisfaction at 3 months [52]. The choice may come down to patient preference: a reusable pessary vs. discrete disposable inserts for specific situations.

Urethral inserts or patches represent another category—for example, a small plug that seals the urethral meatus during activities (removed for voiding). These are less commonly used due to discomfort and UTI risk with prolonged use, but they can be offered for short-term needs (such as during exercise sessions) [53].

Overall, conservative aids do not cure SUI but help manage it, and they play an important role for those who either await definitive treatment or prefer to avoid surgery. It should be acknowledged that evidence quality for devices is moderate (few RCTs), and NICE guidelines note that data are limited. Still, given their low risk, a therapeutic trial of a pessary or insert is reasonable in many cases before proceeding to invasive options [25].

Bulking agents offer a less invasive option by injection of materials (e.g., silicone particles, calcium hydroxyapatite, polyacrylamide hydrogel) into the urethral submucosa to “bulk” the urethral coaptation. Urethral bulking can reduce SUI episodes but usually does not achieve full cure in most patients. A retrospective 7-year study of one bulking agent reported about 67% of women had at least temporary improvement, but over 19% required repeat injections and objective cure rates were low [45]. Bulking is typically reserved for patients with intrinsic sphincter deficiency (ISD) without significant urethral hypermobility, or for those who cannot undergo more invasive surgery. It can also be considered in older or frail patients where surgery risks are high. Complications of bulking are minimal (transient urinary retention or urinary tract infection in a few cases; rare granuloma formation). However, the benefits often diminish over time as the injected material can either migrate or the body’s fibrotic response diminishes. Many women require repeat injections within 1–2 years to maintain the effect [45].

### 3.7. Areas of Limited Evidence

Some conservative therapies have been marketed but lack strong evidence and thus are not widely recommended in guidelines. One example is laser therapy (e.g., fractional CO_2_ or Er:YAG lasers applied vaginally) for SUI. These lasers, used in facial dermatology, have been tried to “rejuvenate” vaginal collagen and support. Early uncontrolled studies suggested symptom improvement, but recent sham-controlled trials showed no significant benefit of vaginal laser over placebo for SUI [54,55]. A systematic review of 5 RCTs concluded that while lasers may lead to some patient-reported improvement, their efficacy does not reach clinically meaningful thresholds, and long-term safety is unclear. Accordingly, in 2022 the FDA and international urogynecological societies issued warnings that laser devices should not be used to treat SUI outside of research settings [56].

Magnetic stimulation (ExMI) was discussed earlier—it is noninvasive and appealing (patient just sits on a chair), but the evidence is mixed. Some RCTs report significant short-term improvement in SUI parameters with ExMI (often combined with PFMT), whereas others find no difference versus sham. A 2023 systematic review by Strojek et al. reported that extracorporeal magnetic innervation (ExMI) may lead to short-term improvements in incontinence episodes and quality of life in women with SUI or mixed UI, but emphasized that the available trials are small, heterogeneous and at risk of bias, so the certainty of evidence is low [29]. In line with this, current guidelines consider magnetic stimulation an experimental or optional adjunct rather than a standard first-line therapy for SUI, and they do not recommend routine use of vaginal laser or radiofrequency devices outside research settings (low-quality evidence; Level C) [13,57].

The range of conservative treatment options for SUI, including PFMT and adjunctive interventions, is summarized in Table 2.

From a cross-modality perspective, pelvic floor muscle training remains the preferred conservative option, while electrical stimulation and tibial nerve stimulation can offer additional benefit in some women but have not demonstrated superiority over well-conducted PFMT. Adjunctive therapies such as acupuncture, pharmacological agents, local estrogen or devices are best reserved for carefully selected patient profiles (e.g., women who cannot adequately perform PFMT or have specific comorbidities), given their more limited evidence base and more variable cost-effectiveness.

### 3.8. Surgical Treatment

Surgical treatment for SUI is typically considered when conservative measures, such as pelvic floor muscle training, lifestyle modifications, and pharmacotherapy, have not yielded satisfactory results. Alternatively, surgical treatment may be contraindicated or initially refused by the patient. The ideal patient for surgical treatment of SUI is one who is not obese (BMI less than 35), has a predominant stress component of SUI, with urethral hypermobility, and does not have prolapse. The current gold standard for surgical intervention is the mid-urethral sling (MUS) procedure, which has demonstrated high success rates and durability. This minimally invasive technique involves the placement of a synthetic mesh tape under the mid-urethra to provide support and prevent urine leakage during physical activities. There are two main types of MUS: retropubic and transobturator. Both variants have shown comparable efficacy, but the choice of approach may depend on patient-specific factors and surgeon preference [58].

For women with intrinsic sphincter deficiency (ISD), the artificial urinary sphincter (AUS) is an effective surgical solution. This highly specialized procedure requires careful patient selection and counseling, as it involves lifelong device management and potential revision surgeries [59].

Another notable surgical option is the autologous fascial sling, which uses the patient ‘s own tissue to create a supportive sling for the urethra. This procedure is particularly beneficial for patients with contraindications to synthetic materials or those who have experienced complications from previous mesh surgeries and is more invasive and requires a longer recovery period [60].

The problem is that surgical treatment is not always accepted by the patient. Sometimes, there may be strong contraindications to any form of surgical intervention (rarely), or the patient may have undergone other interventions to resolve the same condition, which unfortunately failed. Especially in the case of young women with minimal urine or small leakage during extremely intense efforts, surgical treatment may seem exaggerated or involve disproportionately high risks and potential complications compared to the benefits. In these cases, rehabilitation treatment can be extremely effective and sufficient.

### 3.9. Guideline Convergence

It is noteworthy that major guidelines published in the last decade generally agree on a management algorithm. All emphasize conservative management first. The *2017 AUA/SUFU guideline* on female SUI, for example, states that initial treatment may include PFMT and/or an incontinence pessary, and that patient preference should guide therapy selection (Conditional Recommendation) It goes on to recommend mid-urethral sling as the standard surgical approach if conservative measures are insufficient (Strong Recommendation) [10].

The *2018 EAU guidelines* similarly advise offering supervised PFMT as first-line (Grade A evidence) and discussing surgical options (mid-urethral sling) if conservative treatment fails or if the patient desires a more definitive solution [61].

The *2023 CUA guideline* also mirrors this, adding that duloxetine may be offered (with counseling on side effects) to women who decline surgery and have not achieved continence with non-surgical means [57].

This current narrative review’s findings support these guidelines. One area to highlight is that combination therapy can be useful—for instance, PFMT plus topical estrogen in a postmenopausal woman, or PFMT plus weight loss plus pessary in an obese multiparous woman with SUI. Combining modalities addresses multiple contributing factors (anatomical support, muscle strength, tissue trophicity, abdominal pressure loads) and often yields additive improvements. However, from an evidence standpoint, many combination approaches have not been rigorously compared to single therapies, so clinical judgment is needed.

### 3.10. Patient-Centered Care

The optimal management of SUI should be individualized, but opportunities for primary prevention should also be considered across the reproductive life course. Antenatal pelvic floor muscle training (PFMT) has been shown in randomized trials and a Cochrane review to reduce the incidence and severity of urinary incontinence in late pregnancy and the early postpartum period, particularly in primiparous women, and can therefore be recommended as a preventive strategy in women with or without pre-existing leakage [62]. Factors like the patient’s age, general health, severity of SUI, presence of prolapse or urgency symptoms, and personal preferences (including attitude toward devices or surgery) are all important. For example, a young woman postpartum might be best served with PFMT and lifestyle changes, with an expectation of improvement over months as healing occurs. An elderly woman with mild SUI might prefer using pads and doing exercises rather than pursuing surgery, and that is a valid approach as long as she is comfortable. Conversely, a middle-aged woman whose SUI is severe and unresponsive to diligent PFMT might elect to proceed to a sling sooner rather than later—that is appropriate if conservative measures are truly failing her and her quality of life is significantly impacted. In post-menopausal, postpartum and elderly women, pelvic floor muscle training is frequently implemented as part of a combined conservative strategy, together with modalities such as topical estrogen, biofeedback, electrical stimulation or lifestyle interventions. This multimodal approach reflects the multifactorial nature of SUI in these groups, but it also makes it more difficult to isolate the specific effect of muscle training alone, which should be considered when interpreting the available evidence. When SUI coexists with other pelvic floor disorders such as pelvic organ prolapse, anal incontinence or pelvic pain, management should be coordinated within a multidisciplinary pelvic floor team, as treatment priorities and choices (e.g., timing of prolapse surgery, obstetric anal sphincter repair, or combined rehab programs) may differ from isolated SUI. In such cases, PFMT remains a core component but is typically integrated into broader pelvic floor rehabilitation and, when needed, staged surgical planning. In women with mixed urinary incontinence, conservative treatment should target both components: PFMT and lifestyle measures for the stress component, alongside bladder training and, where appropriate, antimuscarinic or β3-agonist therapy for urgency symptoms, before considering invasive options. For refractory mixed UI, procedures such as mid-urethral sling can improve the stress component, whereas neuromodulation or intradetrusor botulinum toxin are reserved for persistent urgency in carefully selected patients. It is important to manage expectations: conservative treatments generally require time and effort to be effective (in contrast to an oral medication that can be taken immediately). Educating patients that improvement may take more than 3 months of training sets realistic timelines and can improve adherence. It is also key to regularly follow up during conservative management to monitor progress; some patients who see minimal initial improvement might be discouraged and abandon therapy if not reassured and guided [4]. Taken together, these considerations support a stepwise, individualized treatment plan based on symptom severity, comorbidities and patient preferences rather than a one-size-fits-all algorithm.

In clinical practice, conservative options are best chosen using a simple, decision-oriented framework that takes into account age, symptom severity, comorbid pelvic floor disorders, menopausal status, prior treatment attempts and patient preferences. PFMT is combined with other modalities according to whether the patient is overweight, postpartum, postmenopausal with GSM, unwilling or unable to perform exercises, or at high surgical risk. Table 3 summarizes how these factors can guide tailoring of conservative strategies to common clinical profiles.

**Figure 3:**
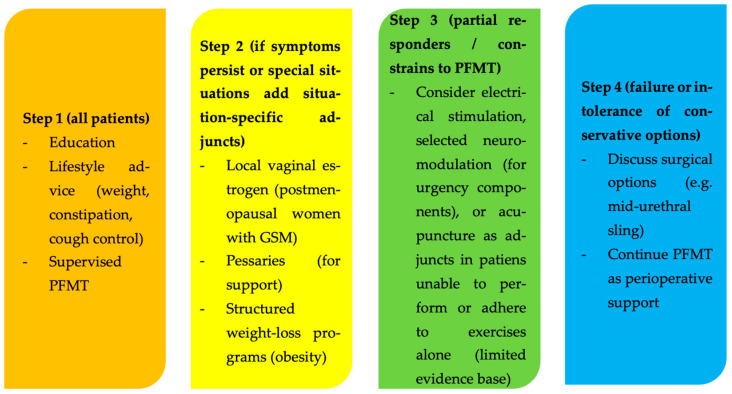
Stepwise conservative management algorithm for female stress urinary incontinence. Step 1 (education, lifestyle advice and supervised PFMT) represents universal first-line treatment for all patients. Steps 2–3 illustrate situation-specific and limited-evidence adjunctive options added when symptoms persist or PFMT is constrained, and Step 4 represents transition to surgical options (e.g., mid-urethral sling) with continuation of PFMT as perioperative support.

To further support clinical application of these findings, a schematic representation of the recommended stepwise approach to conservative management of female SUI has been included (Figure 3). This figure summarizes how core interventions such as PFMT can be combined or sequenced with adjunctive therapies and, when necessary, surgical options, providing a practical visual aid for day-to-day decision-making.

### 3.11. Limitations of Evidence and Future Research

For several conservative interventions, the available evidence is relatively limited and heterogeneous, which makes it difficult to clearly distinguish between insufficient data and possible lack of efficacy. In this context, the absence of robust studies should be viewed with caution rather than interpreted as definite evidence of ineffectiveness, and it underscores the need for further well-designed, adequately powered studies to clarify the role of these therapies. In addition, a formal, study-by-study risk-of-bias assessment was not undertaken, and this is acknowledged as a methodological limitation of the present narrative review.

While PFMT’s efficacy is well-established, there are still unanswered questions about how to optimize it—e.g., the ideal training regimen, the long-term adherence strategies, and how to identify responders versus non-responders early. Further research into behavioral techniques to improve exercise adherence (perhaps leveraging technology or group support) would be valuable. For other conservative treatments, more rigorous trials are needed. Acupuncture, for instance, would benefit from larger multicenter RCTs in Western populations to validate the promising results seen in some Chinese studies. The role of combination therapy (PFMT plus another modality) is also relatively under-studied; most trials isolate one intervention. It is plausible that combination therapy could yield synergistic benefits (as suggested by the meta-analysis where PFMT plus acupuncture yielded better outcomes than PFMT alone), but more evidence is needed to formally recommend combinations. On the surgical front, long-term data (10 or more years) on newer sling types (single-incision slings) and the outcomes of repeat sling surgery versus alternatives (e.g., bulking or fascial sling after a failed synthetic sling) are areas of active investigation. In addition, PFMT has an important adjunctive role before and after surgical treatment of SUI: preoperative training can optimize pelvic floor strength and continence mechanisms, while postoperative PFMT supports recovery, helps manage residual or recurrent leakage and may improve long-term functional outcomes. Recent longitudinal studies and guideline updates also emphasize that mid-urethral slings and other surgical procedures do not guarantee lifelong cure; a proportion of women will experience persistent or recurrent SUI over time, highlighting the need for continued conservative measures and realistic counseling about the possibility of secondary procedures or alternative treatments such as bulking agents or autologous fascial slings [27,57]. Moreover, patient-centered outcomes like activity levels and sexual function after different treatments deserve more attention in trials—curing incontinence is the goal, but preserving or enhancing overall wellness is equally important.

## 4. Conclusions

Conservative management remains the first-line approach for female SUI and should be offered to all women, as pelvic floor muscle training and other guideline-endorsed therapies can significantly reduce leakage and improve quality of life for a substantial proportion of patients. When symptoms persist despite an adequate trial of conservative measures, surgical options—particularly mid-urethral slings—provide well-established, high cure rates and should be discussed within a shared decision-making framework that balances benefits, risks and patient preferences. At the same time, several emerging or adjunctive modalities (such as vaginal laser therapy, routine neuromodulation for pure SUI or certain complementary approaches) are supported by limited or inconclusive evidence and should currently be regarded as investigational, underscoring the need for further high-quality studies that also incorporate patient-centered outcomes.

## Figures and Tables

**Table 1:** Risk Factors Contributing to Urinary Incontinence.

Factor	Description
Advancing Age	Weakened pelvic floor muscles and decreased bladder elasticity with aging
Genetic Predisposition	Hereditary component in some cases
Obstetric History	Multiple pregnancies, vaginal deliveries, complicated childbirth leading to pelvic floor dysfunction
Surgical History	Gynecological and pelvic surgeries (e.g., hysterectomy) altering bladder and urethra support
Smoking	Chronic coughing stresses pelvic floor muscles
Chronic Constipation	Persistent straining weakens pelvic support structures
Menopause	Estrogen deficiency reduces elasticity and strength of urogenital tissues
High Impact Sports	Activities increasing intra-abdominal pressure strain pelvic floor muscles

**Table 2:** Comparative summary of therapies and interventions for stress urinary incontinence (SUI) and related pelvic floor muscle training, highlighting effectiveness, supporting evidence, safety profile, and key limitations based on clinical guidelines and recent reviews. Evidence level was described using guideline-like terminology, with ‘strong’ or ‘Level 1’ referring mainly to systematic reviews/meta-analyses and randomized controlled trials, ‘moderate’ to large observational studies or consistent guideline recommendations, and ‘low’ to small, heterogeneous or indirect studies.

Therapy/Method	Effectiveness	Evidence Strength	Key Benefits	Safety/Tolerability	Notes/Limitations
**Pelvic floor muscle training (PFMT)**	Highly effective; 50–70% symptom reduction or cure	Strong evidence; front-line recommended by NICE, AUA	Improves symptoms significantly; improves postoperative outcomes	Excellent safety profile	Requires proper instruction, training duration/intensity, long-term compliance
**Electrical stimulation (ES)**	Some benefit over no treatment; similar or no significant advantage vs. PFMT	Moderate evidence; no superiority to PFMT	Provides symptom improvement	Generally safe; some discomfort and contraindications (e.g., pacemakers, pregnancy)	Not justified as first-line over PFMT
**PTNS/TTNS (neuromodulation)**	Neuromodulation for overactive bladder/urgency component in mixed UI; not indicated for isolated SUI	Moderate evidence	Reversible, safe option for refractory OAB symptoms	Good patient comfort and ease of application	TTNS preferred over PTNS for comfort
**Acupuncture**	Some improvement; low evidence strength	Low evidence level	Relatively low risk	Safe if performed by qualified practitioners	Not a first-line or standalone therapy; better with PFMT
**Pharmacological (e.g., duloxetine)**	Effective in some; side effects limit use	Evidence level 1 for efficacy but limited by tolerability	Optional adjunct	Side effects common; caution advised	Not routinely recommended due to safety concerns
**Local estrogen therapy**	Recommended for menopausal women with UI and vaginal atrophy	Moderate evidence, Recommendation Grade B	Safe; improves symptoms related to atrophy	Minor side effects (discharge, irritation)	Systemic estrogen contraindicated
**Conservative aids/devices (pessary, Impressa)**	Useful for management, not cure	Moderate evidence	Minimally invasive; device choice may improve symptoms	Minimal risks, some vaginal discharge/ulceration	Trial reasonable before surgery; good for those unable or unwilling to do PFMT
**Bulking agents**	Less invasive, reserved for intrinsic sphincter deficiency	Moderate evidence	Useful for patients unsuitable for surgery	Minimal complications (urinary retention, UTI)	Benefits may gradually decrease
**Laser therapy**	No significant benefit over placebo	Low evidence, warnings against routine use	None established	Not recommended outside research settings	FDA and international societies warn against routine use
**Magnetic stimulation (EXMI)**	Mixed evidence; no routine recommendation	Insufficient evidence (Level C)	Noninvasive, patient-friendly	Unknown benefit, safety profile adequate	Evidence from small, heterogeneous RCTs shows short-term improvement, but long-term and comparative benefit versus PFMT remain unclear (low-quality evidence)

**Table 3:** Decision-oriented selection of conservative therapies for female SUI. This table summarizes key clinical considerations and does not assign formal evidence levels.

Clinical Profile/Key Factors	Preferred First-Line Options	Possible Adjuncts or Alternatives
Young postpartum woman, mild–moderate SUI	Supervised PFMT; lifestyle measures (weight, constipation)	Short-term pessary; postpartum continuation of PFMT programs
Overweight/obese woman with SUI	PFMT plus structured weight-loss and activity program	Pessary; consider duloxetine only if surgery unsuitable and counseled
Postmenopausal SUI with GSM/vaginal atrophy	PFMT plus local vaginal estrogen	Pessary; bulking agents if surgery declined or contraindicated
SUI with mixed UI (urgency/OAB component)	PFMT for stress component; bladder training ± OAB drugs	PTNS/TTNS or SNM for refractory urgency; sling only for stress part
SUI in woman unfit or unwilling for surgery	Intensive supervised PFMT ± ES; pessary	Bulking agents; selected acupuncture or magnetic stimulation (adjunct only)

## Data Availability

No new data were created.

## Abbreviations

The following abbreviations are used in this manuscript:

AUS	Artificial urinary sphincter
AUA	American Urological Association
BMI	Body mass index
CUA	Canadian Urological Association
EAU	European Association of Urology
ExMI	Extracorporeal magnetic innervation
FDA	Food and Drug Administration
GSM	Genitourinary syndrome of menopause
ICS	International Continence Society
ICIQ-SF	International Consultation on Incontinence Questionnaire—Short Form
ISD	Intrinsic sphincter deficiency
LUTS	Lower urinary tract symptoms
MeSH	Medical Subject Headings
MUS	Mid-urethral sling
NICE	National Institute for Health and Care Excellence
OAB	Overactive bladder
PFMT	Pelvic floor muscle training
PTNS	Posterior tibial nerve stimulation
QoL	Quality of life
RCT	Randomized controlled trial
RF	Radiofrequency
SNM	Sacral neuromodulation
SNRIs	Serotonin and norepinephrine reuptake inhibitors
SUI	Stress urinary incontinence
TAS-303	Selective norepinephrine reuptake inhibitor TAS-303
TENS	Transcutaneous electrical nerve stimulation
TTNS	Transcutaneous tibial nerve stimulation
UUI	Urgency urinary incontinence
UI	Urinary incontinence
US	United States (in context of FDA approval)
